# Establishing a nutrition calculation model for catering food according to the influencing factors of energy and nutrient content in food processing

**DOI:** 10.3389/fnut.2024.1388645

**Published:** 2024-04-18

**Authors:** Nan Li, Liangzi Cong, Heng Wang, Yamin Chen, Zhaowei Liu, Mingliang Li, Dong Yang, Huzhong Li, Haiqin Fang

**Affiliations:** ^1^NHC Key Laboratory of Food Safety Risk Assessment, China National Center for Food Safety Risk Assessment, Beijing, China; ^2^School of Biological Science and Technology, University of Jinan, Jinan, China; ^3^Huaiyin District Center for Disease Control and Prevention, Jinan, China; ^4^Key Laboratory of Health Risk Factors for Seafood of Zhejiang Province, Zhoushan Municipal Center for Disease Control and Prevention, Zhoushan, China; ^5^School of Computer and Control Engineering, YanTai University, Yantai, China; ^6^Department of Computer Science, Georgia State University, Atlanta, GA, United States

**Keywords:** catering food, nutritional components, calculation model, raw and cooked ratio, correction factor

## Abstract

**Objective:**

This study aimed to establish an accurate and efficient scientific calculation model for the nutritional composition of catering food to estimate energy and nutrient content of catering food.

**Methods:**

We constructed a scientific raw material classification database based on the Chinese food composition table by calculating the representative values of each food raw material type. Using China’s common cooking methods, we cooked 150 dishes including grains, meat, poultry, fish, eggs, and vegetables and established a database showing the raw and cooked ratios of various food materials by calculating the ratio of raw to cooked and the China Total Diet Research database. The effects of various cooking methods on the nutritional composition of catering food were analyzed to determine correction factors for such methods on the nutritional components. Finally, we linked the raw material classification, raw and cooked ratio, and nutritional component correction factor databases to establish a model for calculating the nutritional components of catering food. The model was verified with nine representative Chinese dishes.

**Results:**

We have completed the construction of an accurate and efficient scientific calculation model for the nutritional composition of catering food, which improves the accuracy of nutrition composition calculation.

**Conclusion:**

The model constructed in this study was scientific, accurate, and efficient, thereby promising in facilitating the accurate calculation and correct labeling of nutritional components in catering food.

## 1 Introduction

With the development of society and the acceleration of the pace of life, an increasing number of people chose to dine out in China (1). The nutritional quality of food supplied provided by the catering industry is of particular concerning, as these foods may account for a significant proportion of the daily food intake, and even of the total intake of the population. However, many consumers are less aware of the energy, fat, and sodium (Na) content of the food provided by restaurants, and excessive consumption of these foods increases the risk of chronic diseases, such as coronary heart disease (2, 3).

The nutrition label of catering food can help consumers understand the nutrient type and content in various dishes and guide them to make reasonable choices according to their own needs to improve their quality of life (4, 5). In December 2020, the National Health Commission of the People’s Republic of China issued “Guidelines for Nutritional Labeling of Catering Food.” This guideline clearly defines the basic labeling content (energy, fat, and Na) and optional labeling content (protein, carbohydrates, sugar, minerals, and vitamins) (6). Since the release of this guideline, catering enterprises have provided nutrient content labels. However, the accuracy of the calculated nutritional components is uncertain. Therefore, it is necessary to establish a scientific and accurate model for calculating and labeling the nutrient content of catering food.

Currently, data from the nutritional composition database of catering food are primarily based on the Chinese food composition tables (7, 8). It is difficult to scientifically classify and calculate some food subcategories with different names in different regions (9), and the nutrient composition of the same food can vary with the maturity of the food materials. Therefore, a scientific nutrient classification database and a raw and cooked ratio database of food materials are necessary for a nutritional composition calculation model. In addition, considering the diverse cooking methods in traditional Chinese food, it is necessary to explore the impact of different cooking methods on nutritional components, in order to determine the differences between the calculated value and the measured value of nutritional components in catering food, and to identify the correction factors for the effects of these cooking methods.

Therefore, we established three databases regarding the raw material classification database, raw-to-ripe ratio of materials, and correction factors. Using computer technology, we linked these databases to our calculation model. Furthermore, we validated the model using nine test dishes and found that the model is scientific and reasonable, indicating its potential in providing technical support for the accurate calculation and correct identification of nutritional components in catering food.

## 2 Materials and methods

### 2.1 Materials

After interviewing with 24 nutrition experts and 18 workers in the catering food industry thrice, we formulated a food sampling plan for different cooking methods (Table 1). We then included 150 samples encompassing the following five categories: grains, meat (chicken, pig, beef, and mutton), fish, eggs, and vegetables (green leafy vegetables and potatoes). The sampling principle followed the requirements of national food safety risk monitoring (10). From different supermarkets, farmers’ markets, and e-commerce platforms (Carrefour supermarkets, local farmers’ markets, and Jing dong online malls) in Beijing, three raw material samples were collected at 2 kg each for each ingredient type (rice, noodles, chicken breast, chicken leg, pork belly, pork legs, brisket, beef tendon, leg of lamb, grass carp, scallops, shrimp, eggs, leafy vegetables, potato). We strictly follow the basic principles of timely sampling, rapid transportation, complete information, and safe storage to collect, transport, preserve, and cook these samples.

**Table 1:** Number of catering food processing plans.

Catering food categories (classified by main ingredients)		Subclass	Number of samples	Processing method	Corresponding dishes
Grain		Rice	9	Steaming, boiling, stir-frying	Steamed rice, boiled rice, stir-fried rice
		Noodles	9	Boiling, stir-frying	Noodles in soup, lao mein, chow mein
Meat	Chicken	Chicken breast	9	Stewing (with soy sauce), deep-frying, stir-frying	Braised chicken breast, fried chicken breast (covered with flour), fried chicken
		Chicken leg	12	Stewing, deep-frying, steaming, roasting	Braised chicken thighs, steamed chicken thighs (cut into pieces and steamed), fried chicken thighs (covered with flour), baked chicken thighs
	Pork	pork belly	12	Steaming, Stewing (with soy sauce), stir-frying, roasting	Braised Pork, Button Pork, Shredded Pork with Green Pepper, Roasted Pork
		Pork legs	12	Steaming, Stewing (with soy sauce), stir-frying, deep-frying	Steamed pork with vermicelli, braised pork, roasted pork, crispy pork
	Beef and mutton	Brisket	9	Stewing (with soy sauce), stir-frying, roasting	Braised beef brisket, roast beef brisket, stir-fried beef brisket
		Beef tendon	9	Stewing (with soy sauce), stir-frying, roasting	Stir-fried beef, roast beef tendon with sauce, braised beef tendon
		Leg of lamb	9	Stewing (with soy sauce), stir-frying, roasting	(Cumin) lamb, roast lamb, braised lamb leg
Fish and shrimp		Grass carp	12	Steaming, Stewing (with soy sauce), stir-frying, roasting	Braised grass carp, fried grass carp, steamed grass carp, grilled grass carp
		Scallops	9	Steaming, Stewing (with soy sauce), deep-frying	Steamed scallops, braised scallops, fried scallop pieces
		Shrimp	9	Steaming, Stewing (with soy sauce), deep-frying	Prawns in oil, steamed prawns, boiled prawns
Egg		Eggs	9	Stewing (with soy sauce), stir-frying	Scrambled eggs with green peppers, boiled eggs, fried eggs
Vegetables		Leafy vegetables	6	Quick-boiling, stir-frying	Vegetable stir-fry with stir-fried greens and boiled greens
		Rootstock (potato)	9	Stewing (with soy sauce), stir-frying, roasting, deep-frying	Hot and sour shredded potatoes, roasted potatoes, french fries (slices), boiled potatoes
Total		150 copies			

### 2.2 Weighing and cooking

After collecting the raw material samples, we processed the food according to the plan with different cooking methods. A total of 150 dishes were cooked. Before cooking, we weighed and recorded the raw materials and seasonings (oil, salt, chicken essence, soy sauce, curd, vinegar, and sugar). The ingredients accurate to 1 g, and the seasonings accurate to 0.1 g. Supplementary Figure S1 shows the weighing procedure. After weighing, we cooked the food, conforming to the traditional Chinese cooking methods, including steaming, boiling (usually with soy sauce), and stir-frying. Supplementary Figure S2 illustrates the cooking process of 150 dishes and some finished dishes. Dishes with different cooking methods were weighed after cooking.

### 2.3 Nutrient composition detection method and quality control

On the basis of the Guidelines for Nutritional Labeling of Catering Foods, we detected fat, protein, carbohydrate and Na in the 150 sample dishes after cooking. Before detection, the inedible parts such as bones/shells were removed and weighed. Fat, protein, ash, and Na were determined according to the relevant national food safety standards (11–14). Carbohydrate content was calculated using the following formula: carbohydrate = total mass – water – ash – protein − fat.

The standard substance, which was used for quality control, was protein/fat in milk powder (FD080-QC) purchased from LGC Technology. Na [GBW10045 (GSB-23)] and ash (RM-18023) in milk powder were both purchased from the Center Testing International Group Co., Ltd. Based on the experimental scheme, the detection results were compared to verify the reliability of the detection method.

### 2.4 Establishment of a nutritional composition calculation model for catering food

In this study, we classified the raw food materials according to the nutritional composition data in the Chinese food composition tables and calculated the representative value of nutrients. Based on this classification, we established a scientific nutritional composition classification database with a wider coverage. Afterward, we selected rice, noodles, beef, pork, mutton, chicken, fish, eggs, potatoes, and green vegetables, which contributed major of nutrients intake in Chinese cooking. Using the most common cooking methods (steaming, boiling, stir-frying, and deep-frying), we cooked 150 Chinese dishes. Thereafter, the nutrients were detected in the laboratory and calculated according to a nutrition calculation formula. Subsequently, we compared the differences between the detected and calculated values of macronutrients and Na in 150 dishes by the various cooking methods. The main effects of the cooking methods on the macronutrients and Na for each ingredient were analyzed, and the correction factors were determined according to the influence of the different cooking methods. In addition, we calculated the actual raw and cooked ratio of different ingredients in 150 dishes and established a raw and cooked ratio database based on the various cooking methods. Finally, we linked the nutrient composition classification, raw and cooked ratio, and correction factor databases to the calculation model. We verified the accuracy of the model‘s calculation results by comparing the calculated and the detected values of nutritional components in nine dishes.

#### 2.4.1 Establishment of a nutritional component classification database for catering food raw materials

**Figure 1:**
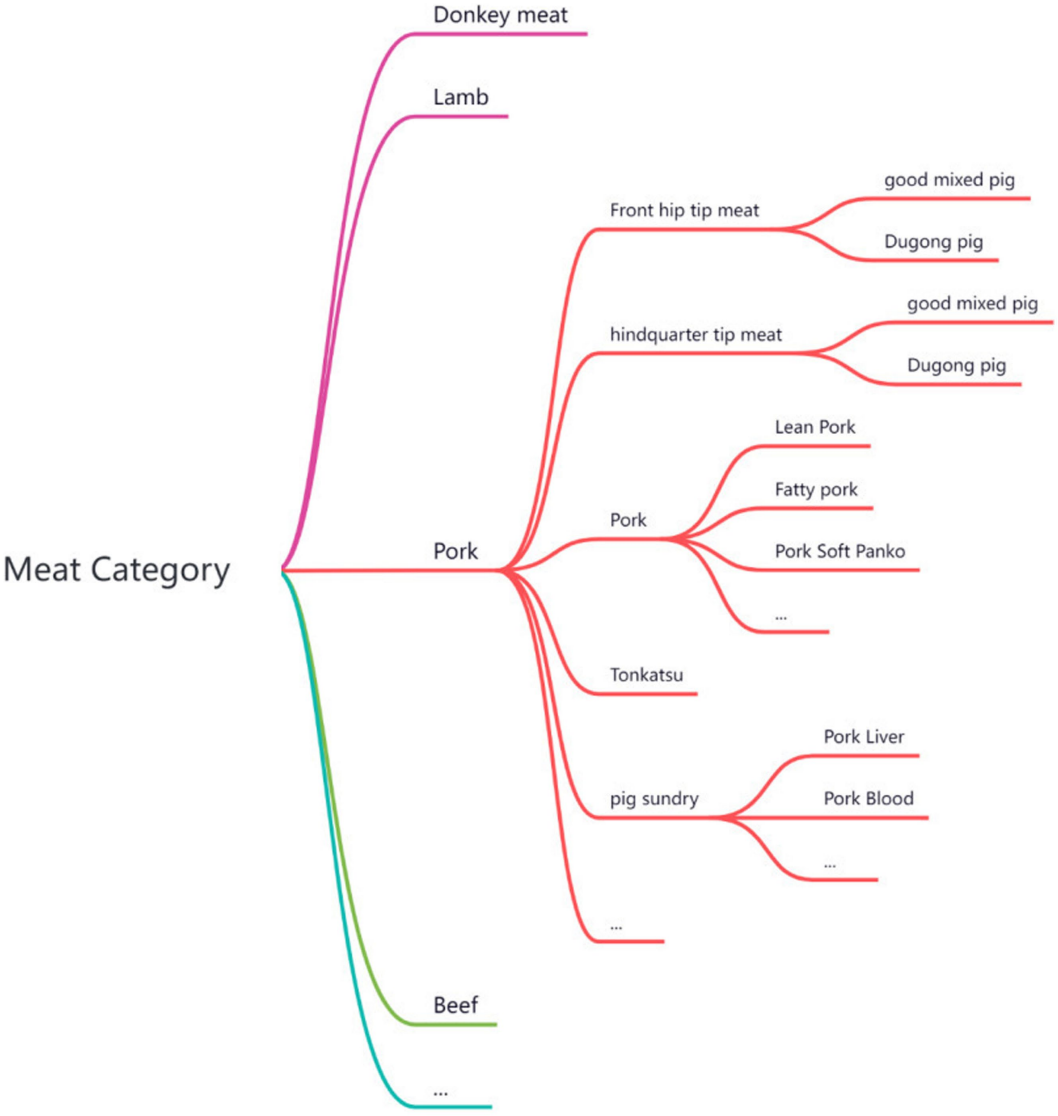
Diagram of the food raw material subcategories.

We first referred the nutritional composition data of food raw materials to the China Food Composition Tables to establish the general category of each food raw material; then, we divided the general category into subcategories (Figure 1). After that, we assigned representative values of nutritional components to each subcategory to ensure the reference of nutritional components for the same or similar categories.

#### 2.4.2 Establishment of a raw and cooked ratio database

To represent the raw and cooked ratio of the food, we calculated the mean value of the raw and cooked ratio of food samples in different provinces included in the Fifth China Total Diet Study (15). First, the grains (rice, flour), poultry meat, livestock meat, fish, shrimp, eggs, and vegetables (green leafy vegetables and potatoes) that contribute greatly to energy and macronutrients (carbohydrates, protein, and fat) were selected and then cooked by steaming, boiling, stir-frying, deep-frying, and roasting. Next, we weighed the raw materials and finished products of the catering food and calculated the actual raw and cooked ratio, thereby establishing a raw and cooked ratio database based on cooking methods.

#### 2.4.3 Establishment of a calculation model program

The calculation of nutritional components of catering food should be to multiply the content of nutritional components in the raw materials by the quality and obtain the amount of nutritional components in each portion. At the same time, it should be considered that the difference of the quality of raw materials and finished products. Therefore, the quality of cooked food should be obtained by combining the ratio of raw and cooked food, and then the value of nutritional components of catering food in the quality of 100 g cooked food should be calculated. At present, the nutritional content of raw materials has a corresponding database, that is, the Chinese food composition table, so it is possible to calculate the nutritional content of catering food through the model.

The calculation model program of the present study was based on Browser/Server architecture. For the calculation of the nutritional value of the catering food, in the back-end code, the list of ingredients to be calculated is used as input, and the list contains the nutrient content information of each ingredient (content per 100 g) and the quality of the ingredients. In the calculation process, first, the list of ingredients is traversed to calculate the content of each nutrient in each ingredient. After that, the calculated nutrient content of each food material is summed according to the type, and obtained the nutrient content of the catering food.

Nutrient calculation formula is:


$$Sum_{\mathrm{Nutrients}}=\overset{n}{\underset{i}{\sum }}\left(\mathrm{Nutrient}s_{i}\times \frac{\mathrm{quantit}y_{i}}{100}\right)$$

Where “n” represents the number of ingredients, “i” represents ingredient i, “Nutrients_i_” represents the nutrient content of the ingredient i, and “quantity_i_” represents the quality of the ingredient i.

The calculation formula of nutrient reference value is:


$$\mathrm{NutrientsNR}V_{\mathrm{act}}=\frac{sum_{\mathrm{Nutrients}}}{\mathrm{NutrientsNR}V_{rec}}$$

In the formula, $\mathrm{NutrientsNR}V_{\mathrm{act}}$ represents the actual nutrient reference value of the catering food, and $\mathrm{NutrientsNR}V_{rec}$ represents the recommended nutrient intake.

The above calculation content pseudo-code can be expressed as:

**Table:** 

Nutrient and reference value calculation
Input: foodlist Output: NutrientsSum and NrvAct N = foodlist.size() NutrientsSum *=* () **for** *i = 0; i ≤ N; i ← i + 1***do** Quantaity = foodlist[i] NutrientsSum = NutrientsSum + (Nutrients ´ quantity/100) **end** NrvAct = NutrientsSum/NrvRec **return** NutrientsSum; NrvAct

Note:

1. In the specific calculation process, Nutrients_i_ can be replaced by any nutrient, which is an abstract expression here.
2. Pseudo-code is only the general calculation process of the program, which is a method to describe the calculation logic.

Based on the above nutrient calculation, the calculation model was designed using two formulas, as shown in Equation 1 and Equation 2. Finally, a calculation model program with user-friendly interface is established. Users could freely enter food information, such as food name, and description, quantity into the system. Food material information was directly linked to the food database in this system. After entering the name of the ingredient, the system would automatically search and caculate for relevant information.

Equation 1 Calculation formula for nutrient composition


(1) $$\begin{array}{l} C\;\left(\mathrm{nutrients,excluding sodium and}\;\mathrm{fat}\right)/100g=\\ \;\frac{\begin{array}{l} \mathrm{Food quality of edible part}\;\left(\mathrm{raw}\right)\;\left(g\right)\times \\ \mathrm{nutrient content of each food}\;\left(g/100g\right)\times \\ \mathrm{ratio of}\;\mathrm{raw-to-cooked} \end{array}}{\mathrm{weight}\;\left(g\right)}\times 100\;g \end{array}$$

Equation 2 Calculation formula for Sodium and fat calculation formula


(2) $$\begin{array}{l} C\;\left(\mathrm{fat}\;\mathrm{or sodium}\right)/100g=\\ \;\frac{\begin{array}{l} \mathrm{Food}\;\mathrm{quality of edible part}\;\left(\mathrm{raw}\right)\;\left(g\right)\times \\ \mathrm{nutrient content}\;\left(g/100g\right)\times \\ \mathrm{ratio of}\;\mathrm{raw-to-cooked}+\\ \mathrm{Cooking oil or salt}\;\left(g\right) \end{array}}{\mathrm{weight}\;\left(g\right)}\times 100g \end{array}$$

#### 2.4.4 Calculation model program correction

It has been reported that nutrients in food are affected by cooking methods (16–19). By practical operation in the present study, we find the difference between the detection values and the calculated values of catering foods under different cooking methods, such as the fat content of roast beef is higher than that of stewed beef of the same piece of beef. After computing the ratio of the calculated and detection values, it is obvious that many ratios were ≤ 0.5 or ≥ 2, indicating a large difference between these two values. This study proposes correction factors under different cooking methods used to calculate the nutritional components.

By identifying the ratio of the calculated and detection values of raw materials for different cooking methods, we established a correction factor database between these values and linked it to the model to correct the model. Equation 3 and 4 show the corrected model.

Equation 3 Corrected calculation model.

Calculation formula for nutrient composition


(3) $$\begin{array}{l} C\;\left(\mathrm{nutrients,excluding sodium and}\;\mathrm{fat}\right)/100g=\\ \;\frac{\begin{array}{l} \mathrm{Food quality of edible part}\;\left(\mathrm{raw}\right)\;\left(g\right)\times \\ \mathrm{nutrient content of each food}\;\left(g/100g\right)\times \\ \mathrm{ratio of}\;\mathrm{raw-to-cooked}\times \mathrm{correction factor} \end{array}}{\mathrm{weight}\;\left(g\right)}\times 100g \end{array}$$

Equation 4 Corrected calculation model.

Sodium and fat calculation formula.


(4) $$\begin{array}{l} C\;\left(\mathrm{fat}\;\mathrm{or sodium}\right)/100g=\\ \;\frac{\begin{array}{l} \mathrm{Food}\;\mathrm{quality of edible part}\;\left(\mathrm{raw}\right)\;\left(g\right)\times \\ \mathrm{nutrient content}\;\left(g/100g\right)\times \\ \mathrm{ratio of}\;\mathrm{raw-to-cooked}\times \mathrm{correction}\;\mathrm{factor}+\\ \mathrm{Cooking oil or salt}\;\left(g\right) \end{array}}{\mathrm{weight}\;\left(g\right)}\times 100g \end{array}$$

Note: The “Food quality of edible part (raw)” in Equations 1—4 refers to the weight of the edible part of the raw material before cooking food. “Nutrient content” refers to the content value of each nutrient in the raw material in the Chinese food composition table, and “weight” is the weight of the food after cooking. In this study, the amount of fat not fully absorbed by food in food or edible oil was also included.

#### 2.4.5 Validation of the corrected model

Based on the common catering food of Chinese residents, we selected and weighed various food raw materials (such as rice, eggs, chicken, pork, zucchini, lettuce, broccoli, etc) and seasonings, and then processed nine dishes. Subsequently, we detected the actual nutritional components (total fat, carbohydrate, protein, and Na) and energy values of these dishes. We used the model before and after correction to calculate these nutritional components and energy values. Thereafter, we compared the difference between the calculated values of the model and the detected values before and after correction.

#### 2.4.6 Data analysis

The detection values of nutrient content in various catering foods were analyzed by IBM SPSS Statistics version 26 (IBM SPSS Inc., Chicago, United States), and the data were statistically analyzed in the form of mean ± standard deviation.

## 3 Results

### 3.1 Raw and cooked ratio calculation results

Generally speaking, as food material matures, their quality and nutrient content will change. In this study, we calculated the raw and cooked ratio of 150 samples and established the raw and cooked ratio database. Table 2 shows the results.

**Table 2:** Calculation results of the raw and cooked ratio of the processed food samples.

Category	Dishes name	Cooking way	Ratio
Rice	Steamed Rice	Steaming	0.49
	Fried Rice	Stir-frying	0.48
Noodles	Noodles in Soup	Boiling	0.84
	Fried Noodles	Stir-frying	0.78
Chicken breast	Braised Chicken Breast	Stewing (with soy sauce)	1.05
	Deep-fried chicken breast (covered with flour)^a^	Deep-frying	1.25
	Fried Chicken	Stir-frying	0.97
Chicken Legs	Braised Chicken Thighs	Stewing (with soy sauce)	1.22
	Steamed chicken thighs (cut into pieces and steamed)^a^	Steaming	0.95
	Fried Chicken Legs (Flour Coated)^a^	Deep-frying	1.08
	Grilled chicken thighs	Roasting	1.43
Pork Five-flower	Braised Pork	Stewing (with soy sauce)	1.28
	Stir-fried Shredded Pork with Green Pepper	Stir-frying	1.05
Pork shank	Steamed Pork in Vermicelli	Steaming	0.65
	Small crispy pork (breaded and fried)	Deep-frying	1.25
	Back-pot meat	Stir-frying	1.12
	Braised Pork	Stewing (with soy sauce)	1.26
Beef Brisket	Braised Beef Brisket	Stewing (with soy sauce)	1.35
	Roast Beef Brisket	Roasting	1.07
	Stir Fried Beef Brisket	Stir-frying	1.16
Beef Tendon	Stir Fried Beef	Stir-frying	1.06
	Roast Beef Tendon with Sauce	Roasting	1.10
	Braised Beef Tendon	Stewing (with soy sauce)	1.34
Leg of lamb	(Cumin) Lamb	Stir-frying	1.21
	Roast Lamb	Roasting	1.24
	Braised Lamb Shank	Stewing (with soy sauce)	1.47
Grass carp	Braised Grass Carp	Stewing (with soy sauce)	0.96
	Deep Fried Grass Carp	Deep-frying	1.11
	Steamed Grass Carp	Steaming	1.01
	Grilled Grass Carp	Roasting	1.11
Hairtail	Steamed hairtail	Steaming	1.09
	Braised hairtail	Stewing (with soy sauce)	0.91
	Deep Fried hairtail Pieces	Deep-frying	1.17
Shrimp	Stewed Prawns	Deep-frying	0.82
	Steamed Prawns	Steaming	1.27
	Boiled Prawns	Stewing (with soy sauce)	1.13
Egg	Scrambled Eggs with Green Peppers	Stir-frying	0.92
	Boiled Eggs	Stewing (with soy sauce)	1.01
	Fried Eggs	Stir-frying	1.14
Pakchoi cabbage	Stir Fried pakchoi cabbage	Stir-frying	1.05
	Quick-boiledpPakchoi cabbage	Quick-boiling	1.00
Potatoes	Spicy and sour shredded potatoes	Stir-frying	1.12
	Fried French fries (slices)	Deep-frying	1.53
	Boiled potatoes	Stewing (with soy sauce)	1.05

### 3.2 Detection results of the sample dishes

Using the detection method, we identified the nutritional components of the 150 samples. Table 3 shows the results.

**Table 3:** Detection value of macronutrients in catering food.

Category	Dishes name	Cooking way	Total fat g/100 g	Protein g/100 g	Sodium g/100 g	Carbohydrate g/100 g
Rice	Rice	Boiling	1.12 ± 0.59	3.16 ± 0.15	0.0014 ± 0.0004	59.73 ± 14.33
	Steamed rice	Steaming	1.22 ± 0.11	3.77 ± 0.28	0.0051 ± 0.0007	53.39 ± 2.45
	Fried rice	Stir-frying	5.97 ± 2.85	3.21 ± 0.44	0.487 ± 0.0954	47.8 ± 6.89
Noodles	Soup noodles	Boiling	1.53 ± 1.31	4.61 ± 0.46	0.0029 ± 0.0002	44.74 ± 1.89
	Fried noodles	Stir-frying	4.35 ± 1.38	6.25 ± 0.26	0.4837 ± 0.0205	53.69 ± 1.54
Chicken breast	Braised chicken breast	Stewing (with soy sauce)	5.14 ± 1.58	24.91 ± 0.21	0.273 ± 0.053	13.9 ± 2.07
	Deep-fried chicken breast (breaded with flour)	Deep-frying	9.4 ± 2.14	29.66 ± 3.23	0.2277 ± 0.0612	13.84 ± 2.47
	Fried Chicken	Stir-frying	7.05 ± 2.16	21.43 ± 3.76	0.1893 ± 0.025	16.4 ± 4.22
Chicken Legs	Braised Chicken Legs	Stewing (with soy sauce)	8.87 ± 2.7	21.21 ± 2.14	0.425 ± 0.0965	14.83 ± 1.64
	Steamed chicken thighs (cut into pieces and steamed)	Steaming	8.13 ± 1.6	21.47 ± 1.34	0.2617 ± 0.01	12.45 ± 0.81
	Fried chicken thighs (coated with flour)	Deep-frying	12.74 ± 0.6	22.27 ± 0.85	0.3687 ± 0.0438	14.84 ± 2.65
	Grilled Chicken Legs	Roasting	9.7 ± 1.42	24.61 ± 0.44	0.8093 ± 0.1361	13.86 ± 1.36
Pork belly	Braised Pork	Stewing (with soy sauce)	40.27 ± 9.79	19.03 ± 1.11	0.3167 ± 0.0898	10.36 ± 5.32
	Steamed pork practice	Steaming	24.6 ± 14.32	18.4 ± 3.63	0.251 ± 0.1191	14.47 ± 3.41
	Stir-fried Shredded Pork with Green Pepper	Stir-frying	31.24 ± 9.89	18.62 ± 3.32	0.4323 ± 0.0498	11.1 ± 1.68
	Grilled Pork	Roasting	35.98 ± 12.02	18.55 ± 4.12	0.24 ± 0.0148	11.47 ± 1.13
Pork Legs	Steamed pork with vermicelli	Steaming	13.42 ± 6.66	14.23 ± 2.79	0.7963 ± 0.042	27.72 ± 2.44
	Small crispy pork (coated with flour and fried)	Deep-frying	15.83 ± 1.68	25.92 ± 4.35	0.6317 ± 0.2183	17.56 ± 3.46
	Pasteurized pork	Stir-frying	34.1 ± 21.23	16.83 ± 4.67	0.5587 ± 0.1042	7.85 ± 8.73
	Braised Pork	Stewing (with soy sauce)	29.88 ± 12.04	21.77 ± 4.91	0.4813 ± 0.099	11.84 ± 0.93
Brisket	Braised Beef Brisket	Stewing (with soy sauce)	24 ± 2.49	22.78 ± 1.5	0.686 ± 0.2842	12.54 ± 2.7
	Grilled Beef Brisket	Roasting	16.11 ± 11.87	18.28 ± 4.82	0.349 ± 0.1615	19.78 ± 13.52
	Stir Fried Beef Brisket	Stir-frying	25.2 ± 11.52	17.5 ± 3.71	0.794 ± 0.1455	11.07 ± 2.85
Beef Tendon	Stir Fried Beef	Stir-frying	11.87 ± 4.65	22.39 ± 1.86	0.9497 ± 0.2829	9.88 ± 4.4
	Grilled Beef Tendon with Sauce	Roasting	6.9 ± 1.38	23.07 ± 1.45	0.4377 ± 0.0941	16.93 ± 0.28
	Braised Beef Tendon	Stewing (with soy sauce)	17.02 ± 7.6	25.4 ± 1.6	0.6823 ± 0.1627	13.63 ± 3.95
Leg of lamb	(Cumin) Lamb	Stir-frying	24.47 ± 11.5	22.17 ± 3.71	0.6925 ± 0.1138	8.79 ± 2.09
	Roast Lamb	Roasting	18.38 ± 1.42	25.27 ± 3.08	0.524 ± 0.0226	9.14 ± 1.74
	Braised Lamb Shank	Stewing (with soy sauce)	19.57 ± 0.21	28.48 ± 1.91	0.577 ± 0.1117	4.63 ± 0.73
Grass carp	Braised Grass Carp	Stewing (with soy sauce)	15 ± 3.73	15.59 ± 0.79	0.7773 ± 0.0917	16.38 ± 2.24
	Fried Grass Carp	Deep-frying	8.39 ± 4.33	21.53 ± 0.54	0.41 ± 0.0806	19.27 ± 1.73
	Steamed Grass Carp	Steaming	3.85 ± 0.94	20.33 ± 2.28	0.6653 ± 0.1049	16.56 ± 2.57
	Grilled Grass Carp	Roasting	8.5 ± 3.45	24.87 ± 1.97	0.571 ± 0.0676	11.68 ± 0.6
Scallops	Steamed Scallop	Steaming	4.1 ± 0.25	21.87 ± 1.56	0.6107 ± 0.0408	14.76 ± 2.11
	Braised Scallops	Stewing (with soy sauce)	8.55 ± 1.55	19.49 ± 1.07	0.7253 ± 0.0804	15.89 ± 2.58
	Deep Fried Scallop Pieces	Deep-frying	14.54 ± 4.87	23.74 ± 0.9	0.6553 ± 0.1562	12.69 ± 2.4
Shrimp	Prawns in oil	Deep-frying	9.24 ± 1.88	21.03 ± 1.45	0.483 ± 0.0759	23.56 ± 2.19
	Steamed shrimp	Steaming	3.02 ± 1.05	23.29 ± 3.08	0.7947 ± 0.1456	12.91 ± 1.49
	Boiled Shrimp	Stewing (with soy sauce)	2.35 ± 0.31	21.03 ± 3.43	0.178 ± 0.0823	13.89 ± 1.16
Eggs	Scrambled eggs with green peppers	Stir-frying	25.4 ± 2.78	11.12 ± 1.38	0.8295 ± 0.1519	8.77 ± 1.38
	Hard-boiled eggs	Stewing (with soy sauce)	11.24 ± 2.37	14.34 ± 1.56	0.1613 ± 0.0164	13.97 ± 1.42
	Fried Eggs	Stir-frying	24.56 ± 2.18	13.9 ± 0.58	0.1788 ± 0.0142	11.65 ± 2.51
Pakchoi cabbage	Stir Fried Pakchoi cabbage	Stir-frying	7.13 ± 2.8	1.98 ± 0.09	0.8767 ± 0.1747	17.89 ± 4
	Quick-boiled Pakchoi cabbage	Quick-boiling	3.72 ± 0.76	1.51 ± 0.31	0.0805 ± 0.0193	22.39 ± 0.63
Potatoes	Hot and Sour Shredded Potatoes	Stir-frying	10 ± 2.32	1.23 ± 0.15	0.6153 ± 0.1569	25.53 ± 1.57
	Baked Potatoes	Roasting	24.57 ± 2.7	2.65 ± 0.79	0.0076 ± 0.0055	29.26 ± 5.79
	French fries (chips)	Deep-frying	7.38 ± 0.44	2.73 ± 0.81	0.0141 ± 0.0071	33.56 ± 1.72
	boiled potatoes	Boiling	3.77 ± 1.04	2.13 ± 0.39	0.5337 ± 0.2191	28.37 ± 1.47

### 3.3 Nutrient composition model calculation results of the sample dishes

Using the established model, we calculated the nutritional components of the 150 sample dishes (Table 4).

**Table 4:** Calculated values of macronutrients of the sample dishes with different cooking methods.

Category	Dish name	Cooking method	Model calculates nutritional value			
			Total fat g/100 g	Protein g/100 g	Sodium g/100 g	Carbohydrate g/100 g
Rice	Rice	Boiling	0.62	3.41	0.0005	37.47
	Steamed rice	Steaming	0.76	3.23	0.0046	35.48
	Fried rice	Stir-frying	10.05	6.98	0.2295	68.91
Noodles	Soup noodles	Boiling	0.06	4.39	0.0210	34.33
	Fried noodles	Stir-frying	15.24	20.27	0.2261	32.26
Chicken breast	Braised chicken breast	Stewing (with soy sauce)	4.77	22.94	0.3808	1.01
	Deep-fried chicken breast (breaded with flour)	Deep-frying	46.52	24.99	0.5104	1.30
	Fried Chicken	Stir-frying	28.63	20.73	0.4245	1.08
Chicken Legs	Braised Chicken Legs	Stewing (with soy sauce)	5.98	12.28	0.2069	0.02
	Steamed chicken thighs (cut into pieces and steamed)	Steaming	6.46	18.52	0.1040	0.03
	Fried chicken thighs (coated with flour)	Deep-frying	18.83	18.59	0.4129	1.30
	Grilled Chicken Legs	Roasting	11.17	26.23	0.9632	4.58
Pork belly	Braised Pork	Stewing (with soy sauce)	30.76	14.40	0.3509	1.14
	Steamed pork practice	Steaming	28.88	15.12	0.2579	0.47
	Stir-fried Shredded Pork with Green Pepper	Stir-frying	27.02	12.08	0.1010	3.47
	Grilled Pork	Roasting	30.68	13.88	0.0930	2.85
Pork Legs	Steamed pork with vermicelli	Steaming	16.23	12.05	0.1672	4.31
	Small crispy pork (coated with flour and fried)	Deep-frying	45.09	28.47	0.6683	10.05
	Pasteurized pork	Stir-frying	23.20	34.17	0.3828	8.28
	Braised Pork	Stewing (with soy sauce)	26.24	15.01	0.1059	6.87
Brisket	Braised Beef Brisket	Stewing (with soy sauce)	23.85	11.16	0.1261	1.09
	Grilled Beef Brisket	Roasting	31.22	17.27	0.1196	2.01
	Stir Fried Beef Brisket	Stir-frying	36.19	17.64	0.2580	0.67
Beef Tendon	Stir Fried Beef	Stir-frying	10.19	21.05	0.1859	0.39
	Grilled Beef Tendon with Sauce	Roasting	17.82	60.10	0.3190	7.70
	Braised Beef Tendon	Stewing (with soy sauce)	8.10	15.74	0.4907	2.04
Leg of lamb	(Cumin) Lamb	Stir-frying	11.11	16.91	0.2043	0.65
	Roast Lamb	Roasting	10.75	16.94	0.1787	0.56
	Braised Lamb Shank	Stewing (with soy sauce)	12.46	24.37	0.1977	0.27
Grass carp	Braised Grass Carp	Stewing (with soy sauce)	9.58	4.72	0.1969	2.06
	Fried Grass Carp	Deep-frying	9.60	7.49	0.1985	6.08
	Steamed Grass Carp	Steaming	4.35	14.33	0.6949	0.42
	Grilled Grass Carp	Roasting	34.22	45.66	1.0224	0.15
Hairtail	Steamed hairtail	Steaming	8.94	38.96	1.8415	7.68
	Braised hairtail	Stewing (with soy sauce)	8.04	5.35	0.1396	1.51
	Deep Fried hairtail Pieces	Deep-frying	8.53	9.92	0.2946	6.46
Shrimp	Prawns in oil	Deep-frying	14.65	15.84	0.4560	14.03
	Steamed shrimp	Steaming	2.71	39.10	0.8164	2.66
	Boiled Shrimp	Quick-boiling	2.21	42.11	0.7947	2.63
Eggs	Scrambled eggs with green peppers	Stir-frying	11.07	6.71	0.4120	5.84
	Hard-boiled eggs	Stewing (with soy sauce)	6.52	15.22	0.1891	5.43
	Fried Eggs	Stir-frying	32.66	9.70	0.0767	0.81
Pakchoi cabbage	Stir Fried Pakchoi cabbage	Stir-frying	8.76	1.10	0.8042	2.21
	Quick-boiled Pakchoi cabbage	Quick-boiling	0.00	1.23	0.1210	2.47
Potatoes	Hot and Sour Shredded Potatoes	Stir-frying	29.70	5.63	1.2167	47.70
	Baked Potatoes	Roasting	3.21	1.74	0.0043	14.78
	French fries (chips)	Deep-frying	16.50	1.45	0.0041	12.32
	Boiled potatoes	Stewing (with soy sauce)	4.20	0.76	0.0361	5.97

### 3.4 Model calibration

According to the screening principle of the ratio of the calculation value to the detection value of ≤0.5 or ≥ 2, we corrected the calculation models of the nutritional components. Table 5 enumerates the examples of specific corrections.

**Table 5:** Ratio of the calculation value to the detection value^a^.

Category	Dish name	Cooking method	Ratio			
			Total fat	Protein	Sodium	Carbohydrate
Rice	Rice	Boiling	0.55	1.08	0.36*	0.63
	Steamed rice	Steaming	0.62	0.86	0.90	0.66
	Fried rice	Stir-frying	1.68	2.17*	0.47*	1.44
Noodles	Soup noodles	Boiling	0.04*	0.95	7.24*	0.77
	Fried noodles	Stir-frying	3.50*	3.24*	0.47*	0.60
Chicken breast	Braised chicken breast	Stewing (with soy sauce)	0.93	0.92	1.39	0.07*
	Deep-fried chicken breast (breaded with flour)	Deep frying	4.95*	0.84	2.24*	0.09*
	Fried Chicken	Stir-frying	4.06*	0.97	2.24*	0.07*
Chicken Legs	Braised Chicken Legs	Stewing (with soy sauce)	0.67	0.58	0.49*	0.00*
	Steamed chicken thighs (cut into pieces and steamed)	Steaming	0.79	0.86	0.40*	0.00*
	Fried chicken thighs (coated with flour)	Deep frying	1.48	0.83	1.12	0.09*
	Grilled Chicken Legs	Roasting	1.15	1.07	1.19	0.33*
Pork belly	Braised Pork	Stewing (with soy sauce)	0.76	0.76	1.11	0.11*
	Steamed pork practice	Steaming	1.17	0.82	1.03	0.03*
	Stir-fried Shredded Pork with Green Pepper	Stir-frying	0.86	0.65	0.23*	0.31*
	Grilled Pork	Roasting	0.85	0.75	0.39*	0.25*
Pork Legs	Steamed pork with vermicelli	Steaming	1.21	0.85	0.21*	0.16*
	Small crispy pork (coated with flour and fried)	Deep frying	2.85*	1.10	1.06	0.57
	Pasteurized pork	Stir-frying	0.68	2.03*	0.69	1.06
	Braised Pork	Stewing (with soy sauce)	0.88	0.69	0.22*	0.58
Brisket	Braised Beef Brisket	Stewing (with soy sauce)	0.99	0.49*	0.18*	0.09*
	Grilled Beef Brisket	Roasting	1.94	0.94	0.34*	0.10*
	Stir Fried Beef Brisket	Stir-frying	1.44	1.01	0.32*	0.06*
Beef Tendon	Stir Fried Beef	Stir-frying	0.86	0.94	0.20*	0.04*
	Grilled Beef Tendon with Sauce	Roasting	2.58*	2.61*	0.73	0.45*
	Braised Beef Tendon	Stewing (with soy sauce)	0.48*	0.62	0.72	0.15*
Leg of lamb	(Cumin) Lamb	Stir-frying	0.45*	0.76	0.30*	0.07*
	Roast Lamb	Roasting	0.58	0.67	0.34*	0.06*
	Braised Lamb Shank	Stewing (with soy sauce)	0.64	0.86	0.34*	0.06*
Grass carp	Braised Grass Carp	Stewing (with soy sauce)	0.64	0.30*	0.25*	0.13*
	Fried Grass Carp	Deep frying	1.14	0.35*	0.48*	0.32*
	Steamed Grass Carp	Steaming	1.13	0.70	1.04	0.03*
	Grilled Grass Carp	Roasting	4.03*	1.84	1.79	0.01*
Hairtail	Steamed hairtail	Steaming	2.18*	1.78	3.02*	0.52
	Braised hairtail	Stewing (with soy sauce)	0.94	0.27*	0.19*	0.10*
	Deep Fried hairtail Pieces	Deep frying	0.59	0.42*	0.45*	0.51
Shrimp	Prawns in oil	deep frying	1.59	0.75	0.94	0.60
	Steamed shrimp	Steaming	0.90	1.68	1.03	0.21*
	Boiled Shrimp	Quick-boiling	0.94	2.00*	4.46*	0.19*
Eggs	Scrambled eggs with green peppers	Stir-frying	0.44*	0.60	0.50*	0.67
	Hard-boiled eggs	Stewing (with soy sauce)	0.58	1.06	1.17	0.39*
	Fried Eggs	Stir-frying	1.33	0.70	0.43*	0.07*
Pakchoi cabbage	Stir Fried Pakchoi cabbage	Stir-frying	1.23	0.56	0.92	0.12*
	Quick-boiled Pakchoi cabbage	Quick-boiling	0.00*	0.81	1.50	0.11*
Potatoes	Hot and Sour Shredded Potatoes	Stir-frying	2.97*	4.58*	1.98	1.87
	Baked Potatoes	Roasting	0.13*	0.66	0.57	0.51
	French fries (chips)	Deep frying	2.24*	0.53	0.29*	0.37*
	Boiled potatoes	Stewing (with soy sauce)	1.11	0.36*	0.07*	0.21*

a Ratio = Model values of nutritional components / Detection values of nutritional components.

^*^The ratio between the caculation value and the detection value is ≤ 0.5 or ≥.

### 3.5 Validation results of the corrected model

**Figure 2:**
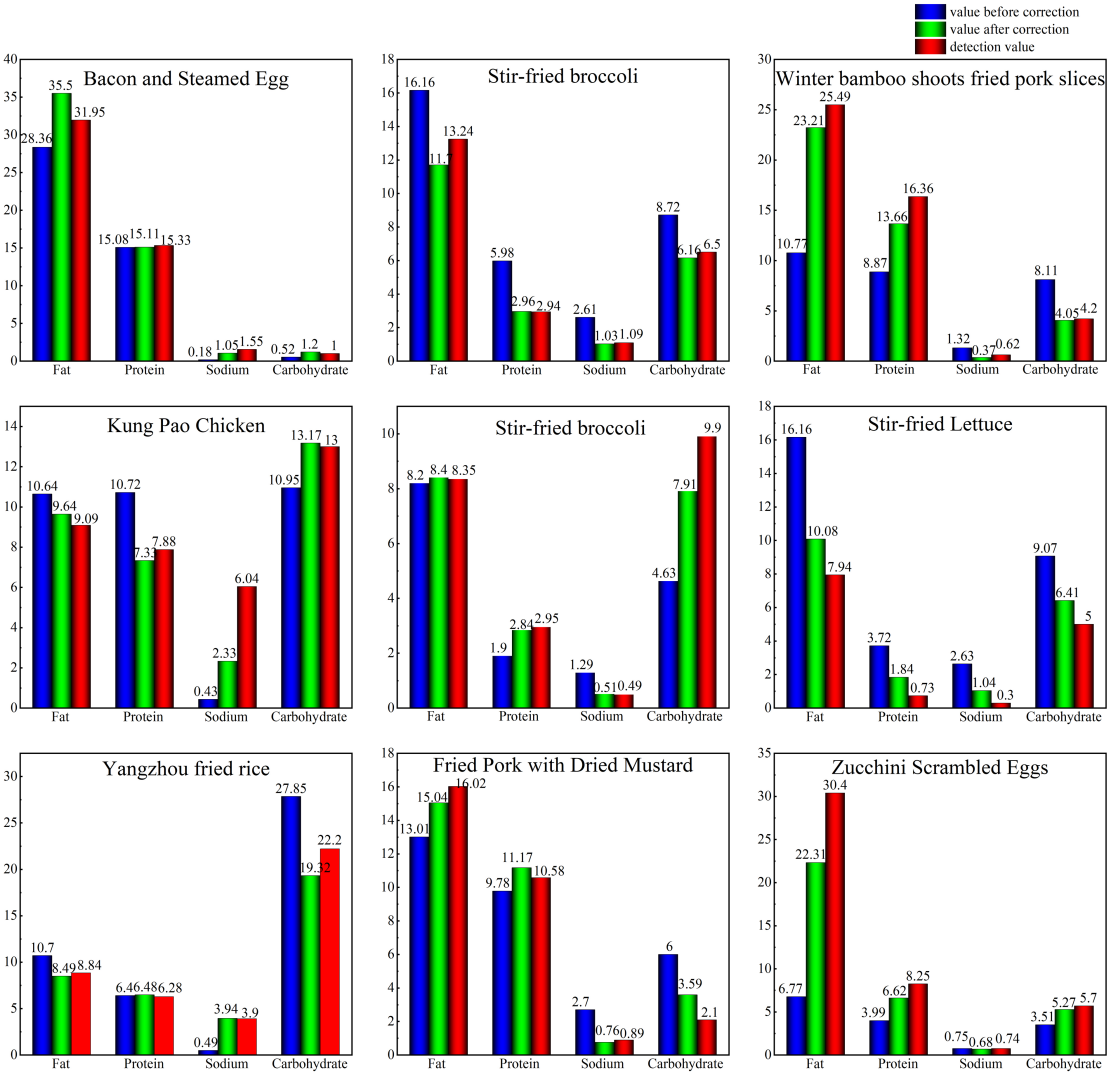
Comparison of the calculated and detected values of the nutrients found in nine dishes.

Using the model before and after correction, we calculated the nutritional components and energy values of nine selected dishes and compared their calculated and detected values. Figure 2 illustrates the results.

## 4 Discussion

### 4.1 Construction of a raw material classification database

The nutritional components of catering food raw materials have a fundamental and direct impact on the calculation of the nutritional components of dishes (9). Therefore, a professional nutritional component database is needed for the correct labeling of catering food. Currently, the nutritional composition database of catering food primarily refers to the China Food Composition Tables 2002 and 2004 (7, 8). However, considering the significant differences in China’s geographical environment, the types and nutritional characteristics of raw food materials are diverse. For example, the same type of raw food materials may have different varieties or names. Considering the different environments in different provinces, the same raw materials may have different nutritional components, making it difficult to include the entire catering ingredient in the composition table. This study established a scientific classification database of catering food raw materials. The average values of each nutrient in each type of food ingredients were calculated as the representative values of each nutrient in this type of food raw materials. These representative values were then used to replace the new varieties of this food type, thereby greatly improving the accuracy of the calculation of the catering food’s nutritional components.

### 4.2 Effect of cooking on nutritional components

Based on different food matrices, different cooking methods have different effects on various nutritional components in catering food. For carbohydrate-rich cereal and potato foods, boiling and stir-frying have a greater impact on their carbohydrate content, deep-frying had the greatest impact on their fat, and stewing and stir-frying had a greater impact on protein. Relevant results have also been reported in previous studies. For example, one study found that three cooking methods, namely, steaming, boiling, and deep-frying, can lose starch and reduce sugar in sweet potato, and the degree of loss from high to low was observed in deep-frying, boiling, and steaming (20). Furthermore, when food is deep-fried or stir-fried, the protein is severely damaged (21, 22).

For fish, meat, and eggs with high fat content, deep-frying affects fat the most, steaming greatly affects protein, and stewing and stir-frying mostly affect carbohydrates. Fat reportedly becomes seriously damaged when the oil temperature is excessively high in the process of deep-frying or stir-frying (23, 24). Compared with steaming and stir-frying, boiling alone can maintain a higher content of amino acids and fatty acids, but it can also induce the production of saturated fatty acids (25). However, cooking studies on foal meat from the Galicia Mountains revealed that steaming and stewing did not affect the total essential amino acids and nonessential amino acids (26). For vegetables, stir-frying affects the nutrients the most. Therefore, for traditional Chinese food, boiling, stir-frying, and deep-frying clearly affect the nutritional components of catering food.

### 4.3 Establishment of a calculation model

It is very difficult to identify the nutritional information of catering food, because the nutritional components are influenced by the raw materials and processing methods, they are difficult to be accurately calculated, and the cost of detection is too high. Through the classification database, the practical problem of some raw materials do not have nutrient composition data in the Food Composition Table is solved, and all most dishes can be included in the model calculation. After the influencing factors in the processing of catering food are corrected, the data calculated by the model are closer to the true value of the dishes. In this study, we successfully constructed a scientific, accurate, and efficient calculation model for the nutritional components, indicating its potential in providing technical support for the accurate calculation and correct identification of nutritional components in catering food. Accurate nutrition information identification will help consumers make scientific choices and maintain physical health.

### 4.4 Limitations of the study

This model established in this study improved the accuracy of calculation by taking into account as many key influential factors to reduce the errors. However, this study has some limitations. Chinese food is extremely rich in raw materials and cooking methods. This model only investigates and corrects for the most common cooking methods through the most commonly consumed meat (pig, beef, sheep, chicken, fish) and cereals (rice), which are only a small part of Chinese food. So we should further improve this model by multiple verification and relevant data supplement, analyze each influencing factor on the nutrition content of foods for catering.

## 5 Conclusion

Catering food nutrition labeling can help consumers identify the type and content of nutrients in dishes. A scientific, accurate, and efficient calculation model is the key to accurately calculate and label catering food nutrients. In this study, we established three complete databases of food classification, raw and cooked ratio, and correction factors according to different cooking methods. Using computer technology, we linked these databases to the calculation model. Ultimately, we were able to develop a scientific, accurate, and efficient calculation model of the nutritional components of catering food to provide support for the accurate calculation and correct identification of nutritional components.

## Data availability statement

The original contributions presented in the study are included in the article/Supplementary material, further inquiries can be directed to the corresponding authors.

## Author contributions

NL: Data curation, Methodology, Writing – original draft. LC: Formal analysis, Writing – original draft. HW: Validation, Writing – original draft. ZL: Formal analysis, Writing – review & editing. ML: Software, Writing – original draft. DY: Software, Writing – original draft. HL: Conceptualization, Writing – review & editing. HF: Conceptualization, Supervision, Writing – review & editing. YC: Formal analysis, Writing – original draft.
