# Supplementary material for: Establishing a nutrition calculation model for catering food according to the influencing factors of energy and nutrient content in food processing
Source: Front Nutr. 2024 Apr 18;11:1388645. doi: 10.3389/fnut.2024.1388645 (PMC11063351; doi:10.3389/fnut.2024.1388645)
Supplement: Supplementary file 1 [file Image_1.pdf]

Supplementary Material

Establishing a nutrition calculation model for catering food according to the influencing factors of energy and nutrient content in food processing

Nan Li [1,2,†], Liangzi Cong [3,†], Heng Wang[4,†], Zhaowei Liu[5], Mingliang Li[5], Dong Yang[6], Huzhong Li [1,*] and Haiqin Fang [1,*]

*** Correspondence:** Haiqin Fang: fanghaiqin@cfsa.net.cn Huzhong Li: lihuzhong@cfsa.net.cn

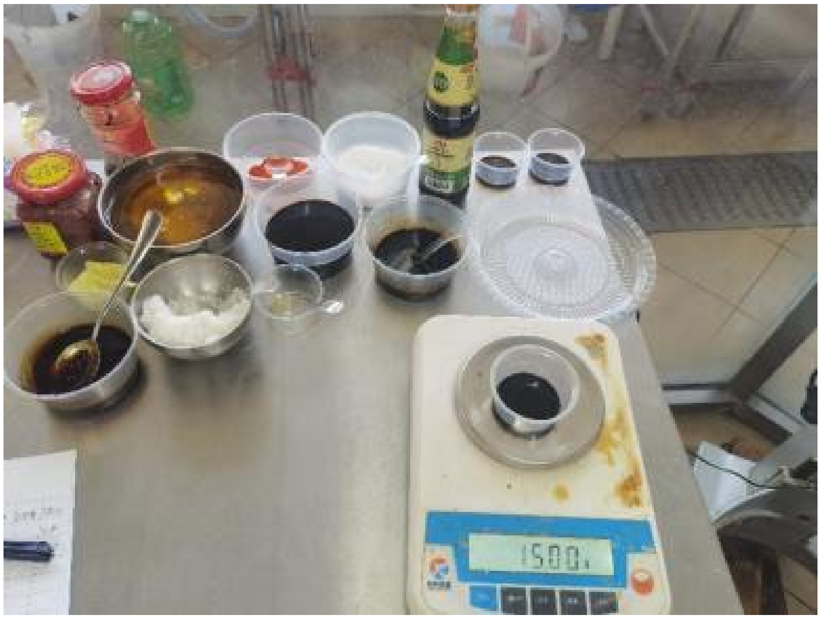

Supplementary Figure 1. Weighing of raw materials and seasonings.

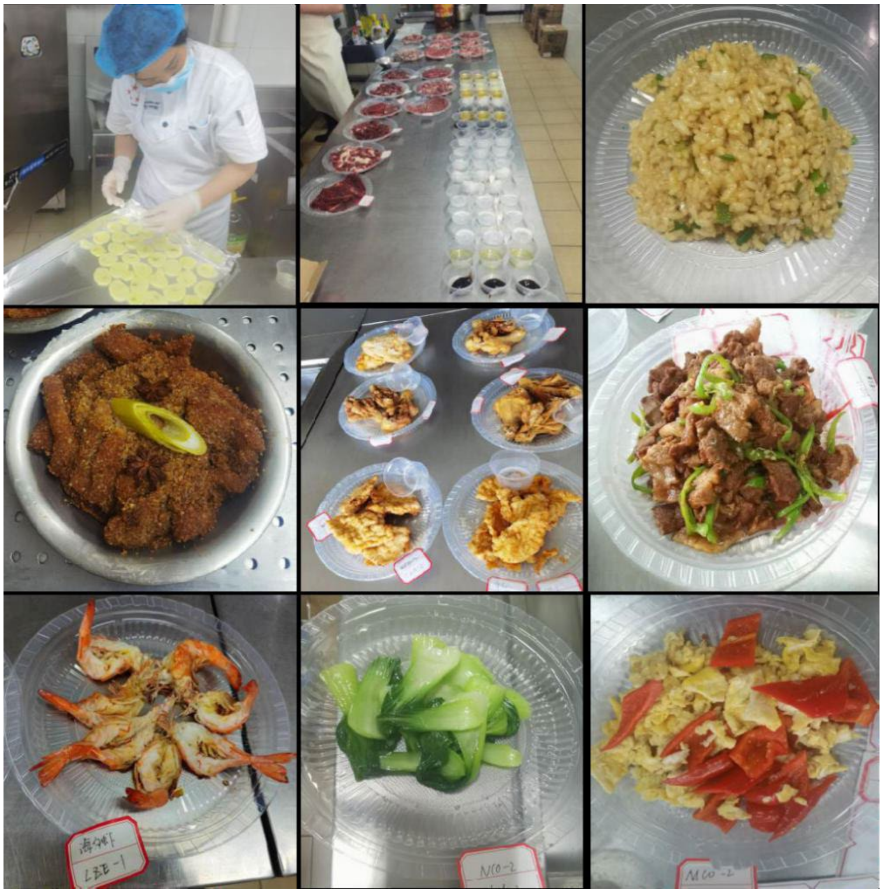

Supplementary Figure 2. Cooking process of 150 dishes and some of the finished dishes.
